# 

**Published:** 1890-08

**Authors:** 


CONTENTS—AUGUST, 1890.—VOL. VI., NO. 2.

ORIGINAL ARTICLES— PAGE.
Septicæmia. Wm. Boll, M. D. . . . 51
A Remarkable Suicide. F. S. White, M. D. . . . 56
A Case of Psoas Abscess, Mistaken During Life for Hernia. J. L. Cunningham, M. D. . . . 58

CORRESPONDENCE—
Letter from London. E. J. Beall, M. D. . . . 61
Archives of the Medical Department of the C. S. Army. Dr. S. H. Stout . . . 68
A Case of Psoas Abscess, etc. Dr. J. L. Cunningham . . . 71

SOCIETY NOTES—
Obituaries of the late Drs. Carothers and Dr. Tyler . . . 72
Resolutions of Delta County Medical Society . . . 77

EDITORIAL—
Finances of the Texas State Medical Association . . . 79
Capital Punishment—The Kemmler Case . . . 83
Notice to Medical Officers, etc . . . 86

MEDICAL NEWS AND MISCELLANY—
Many Important Items of News . . . 86

BOOK NOTICES—
Reviews of new books . . . 92

PUBLISHER'S NOTES—
Calling attention to important matters . . . 93

